# Proton pump inhibitor use is not independently associated with colonic diverticulosis in an asymptomatic screening population

**DOI:** 10.1038/s41598-026-37547-2

**Published:** 2026-01-26

**Authors:** Andreas Völkerer, Sarah Wernly, Georg Semmler, Maria Flamm, Mathias Ausserwinkler, Gabriele Koch, Nikolaus Götz, Hannah Hofer, Elmar Aigner, Christian Datz, Bernhard Wernly

**Affiliations:** 1 Department of Internal Medicine, General Hospital Oberndorf, Teaching Hospital of the Paracelsus Medical University, Salzburg, Austria; 2 Division of Gastroenterology and Hepatology, Department of Medicine III, Medical University of Vienna, Vienna, Austria; 3 Family Medicine and Preventive Medicine, Center for Public Health and Healthcare Research, Institute of General Practice, Paracelsus Medical University, Salzburg, Austria; 4 Department of Internal Medicine, Elisabethinen Hospital Klagenfurt, Klagenfurt, Austria; 5 Department of Internal Medicine, General Hospital, St. Vinzenz Zams, Zams, Austria; 6 First Department of Medicine, University Clinic Salzburg, Paracelsus Medical University Salzburg, Salzburg, Austria

**Keywords:** Diverticulosis, Diverticular disease, PPI, Microbiome, Gastrointestinal diseases, Drug regulation

## Abstract

Proton pump inhibitors (PPIs) are widely used medications that alter gut microbiota. Given the high prevalence of colonic diverticulosis and its increasing incidence in younger populations, we investigated whether PPI use is associated with diverticulosis prevalence in an asymptomatic screening population. This retrospective observational study analyzed data from 6,153 asymptomatic individuals undergoing colorectal cancer screening in Austria. Colonoscopies assessed diverticulosis presence, while PPI use was determined via structured medical history. Statistical analyses, including Poisson regression models and sensitivity analyses, were conducted to evaluate the association between PPI use and diverticulosis, with adjustments for confounding factors such as age, sex, BMI, comorbidities, and lifestyle characteristics. Among 6,153 participants, 37% were found to have diverticulosis, with a significantly higher prevalence observed in PPI users (48%) compared to non-users (36%, *p* < 0.001). PPI users were generally older, had a higher BMI, and were more likely to have cardiometabolic comorbidities. Univariate analysis demonstrated a significant association between PPI use and diverticulosis (RR 1.326, 95% CI: 1.199–1.476, *p* < 0.001). However, this association was not sustained in multivariable models adjusted for age, sex, BMI, comorbidities, and lifestyle factors, indicating that the observed relationship is likely attributable to confounding rather than a direct causal effect. In this large screening cohort, the initially observed association between PPI use and diverticulosis was likely attributable to confounding. These findings suggest that PPI use is not independently associated with diverticulosis.

## Introduction

This retrospective observational study aimed to explore the relationship between proton pump inhibitor (PPI) use and the prevalence of colonic diverticula within an asymptomatic screening population.

Gastroprotective drugs have been developed to shield the mucosal lining and support the healing of injuries in the upper gastrointestinal tract. Since their introduction, PPIs have gradually become the leading treatment for acid-related conditions. PPIs are commonly used for treating gastric and duodenal ulcer disease, preventing ulcer complications during NSAID use, managing ulcer bleeding, treating both erosive and non-erosive gastroesophageal reflux disease, addressing functional dyspepsia, and eradicating Helicobacter pylori in combination with antibiotics^1^. Over more than two decades, PPIs have maintained an excellent safety record, but their widespread use has raised concerns about potential short- and especially long-term effects. Long-term PPI use, often defined as use longer than a year, has been associated with chronic kidney disease, malabsorption of important nutrients such as calcium, vitamin B12, iron, and magnesium, bone fractures, enteral and pulmonary infections, and even increased overall mortality in patients with pre-existing conditions^2–4^. Moreover, a significant impact of PPIs on the gut microbiome has been observed^5,6^.

This could be particularly relevant in the context of diverticulosis, a prevalent condition where small protrusions, known as diverticula, form in the wall of the intestine. These diverticula can affect different layers of the intestinal wall depending on where they are located and are one of the most common changes seen in the colon^7^. The location of diverticular formations varies by ancestry, with diverticula predominantly found in the left colon, especially the sigmoid colon, in individuals of white ethnicity in the Western world, while individuals of Asian descent primarily develop diverticula in the right colon, irrespective of sex, age, or geographic region^8,9^. Focusing on western civilization this condition frequently remains asymptomatic^7,10^, with approximately 10% of individuals under 40 and 50–70% of those over 80 being affected without noticeable symptoms^11^. Nevertheless, its prevalence has escalated, now impacting 50% of those over 60, accompanied by a marked increase in incidence among younger age groups^12^. The classification of asymptomatic diverticulosis as a distinct disease remains debated^13^. However, it is recognized that symptomatic diverticular disease is categorized into two types: symptomatic uncomplicated diverticular disease (SUDD) and symptomatic complicated disease, which includes acute diverticulitis and diverticular hemorrhage^14^. This affects about 10%-25% of patients, who may eventually develop symptoms such as symptomatic uncomplicated diverticular disease (SUDD) or, in more severe cases, diverticulitis (4%), perforation, and bleeding.

Diverticulosis is influenced by genetics^15^, age^16^, cardiometabolic risks^17^, and diet, including alcohol^18^. Recent evidence questions the protective role of a high-fiber diet^19^.

While no direct link between the development of diverticula and PPI use has been studied so far, various studies have investigated an association with diverticulitis and severity of diverticular disease^20,21^ and also explored the role of the intestinal microbiome in the development of diverticula. Up to now fecal and mucosa-associated microbiome in diverticular disease do not show clear alterations related to disease formation or progression, with results being inconsistent across different studies^22,23^. Establishing causality in this context is particularly challenging due to the high risk of confounding.

Due to its rising global prevalence and the occasional yet troublesome complications it can cause, the prevention of diverticulosis has become increasingly important^10^. This growing number of affected individuals also presents a significant challenge to healthcare systems, leading to non-negligible costs^24^.

With PPI use on the rise and diverticulosis becoming more common, we aim to investigate a potential link between these widely used medications and colonic diverticula in an asymptomatic population. By accounting for confounding factors in a representative cohort, this study provides an opportunity to critically assess and, if necessary, challenge this association, offering relevant insights for clinical practice and public health.

## Methods

### Study population

This retrospective cohort study was conducted at the General Hospital Oberndorf near Salzburg, Austria, and evaluated colorectal cancer screening within an opportunistic prevention program recommended in Austria from the age of 45. Participants were recruited through the Salzburg Colon Cancer Prevention and Intervention initiative (SAKKOPI) and comprised asymptomatic individuals who underwent screening at a single center between January 2007 and March 2020. Participation was initiated either through preventive counselling by primary care physicians or via self-referral following public awareness campaigns and local media outreach. Referral by a physician did not indicate the presence of gastrointestinal symptoms but functioned solely as an access pathway to the screening program. All examinations were fully covered by health insurance, ensuring barrier-free access.

To better characterize the study setting and to facilitate interpretation of the study cohort, official population statistics for the federal state of Salzburg at the end of the study period (2021) were used for descriptive purposes only. At that time, Salzburg comprised 560,710 inhabitants. Of these, 268,971 individuals (48.0%) were aged ≥ 45 years and therefore constituted the eligible source population for colorectal cancer screening. This population included 161,365 individuals aged 45–65 years (49% men, 51% women) and 107,606 individuals aged > 65 years (44% men, 56% women). Overall, individuals aged ≥ 45 years comprised 46.7% men and 53.3% women, reflecting a balanced sex distribution in the 45–65-year group and a predominance of women in the > 65-year group. As the screening program was designed as an open, opportunistic, population-based initiative without a predefined sampling frame, an exact participation rate of the eligible source population could not be calculated^25^.

**Fig. 1:**
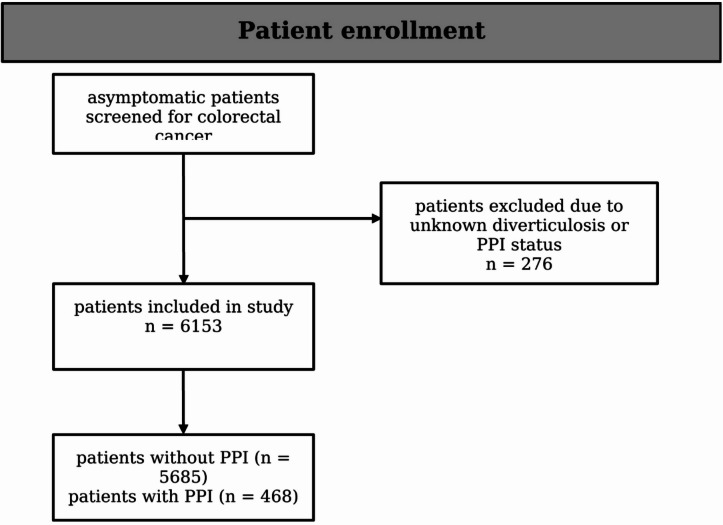
Flow diagram of patient enrollment.

A total of 6153 participants were included in the study. Patients who showed symptoms indicative of colorectal cancer during screening, lacked comprehensive clinical or laboratory information, or did not fully complete the medical history questionnaire were not included in the study. Furthermore, participants whose colonoscopies did not comply with international standards or failed to meet the required performance criteria were also excluded. Individuals with a known history of colorectal cancer, prior treatment for colorectal neoplasia, or medical conditions that made colonoscopy unsafe were not included either. The study also excluded those who declined follow-up or did not provide informed consent. This cohort has been thoroughly detailed in earlier investigations^26^. The process of patient enrollment is illustrated in Fig. 1.

### Patient assessment

Patients underwent a two-day examination process. On the initial day vital signs were monitored and comprehensive clinical evaluations were conducted. Clinical and laboratory data were gathered from all participants, who also completed a questionnaire detailing their medical history and their family. Body mass index (BMI) was calculated according to the standards set by the World Health Organization (WHO), and arterial hypertension was classified following the guidelines provided by the European Society of Cardiology (ESC) for hypertension management^27^. Smoking habits were categorized as “ever smokers” and “active smokers” based on self-reported information. The criteria for diagnosing metabolic syndrome were aligned with the consensus established by the IDF/AHA/NHLBI^28^.

The second day was specifically reserved for conducting colonoscopies, which were exclusively performed by seasoned endoscopists. Sedation during the procedure was administered using either Propofol alone or in combination with Midazolam. All biopsies and resected lesions were examined histopathologically and classified according to their macroscopic and microscopic characteristics.

### Assessment of diverticulosis

A colonoscopy was performed following international standards, ensuring that all relevant performance benchmarks were satisfied^29^. Based on the results, patients were categorized into one of the following groups: ‘no diverticulosis,’ ‘left-sided diverticulosis,’ ‘right-sided diverticulosis,’ or ‘pandiverticulosis’.

### Assessment of PPI intake

Assessment of PPI intake was based on a structured medical history documenting whether patients were receiving PPI therapy at the time of examination. Information on the indication for PPI use and the duration of therapy was not collected. Moreover, the specific PPI compound was not systematically recorded; therefore, compound-specific or potency-related differences between PPI agents could not be analyzed.

### Statistical analysis

Continuous data are presented as median ± interquartile range (IQR) and compared using the Mann–Whitney U test to ensure consistency across all parameters, regardless of distribution. Categorical variables are presented as frequencies (percentages) and were examined using the chi-square test. All statistical tests were two-tailed, with a significance level set at *p* < 0.05. The primary outcome of interest was the presence of diverticulosis. PPI intake was analyzed as the exposure variable in relation to diverticulosis. We performed a statistical analysis using a Poisson regression model to evaluate the association between diverticulosis and the intake of PPIs (proton pump inhibitors). Poisson regression with robust variance estimation was used to model the association between PPI use and diverticulosis, as this approach allows for the direct estimation of prevalence ratios in cross-sectional studies with binary outcomes. Given the relatively high prevalence of diverticulosis in our cohort, odds ratios derived from logistic regression would diverge from prevalence ratios and may be misinterpreted as an overestimation of the relative effect. Therefore, Poisson regression with robust standard errors was chosen to improve interpretability of effect estimates. Initially, a univariate analysis and a sensitivity analysis were conducted. Sensitivity analyses were performed for sex, age (above and below 55 years), obesity (BMI > 30), diabetes (no diabetes, prediabetes, manifest diabetes) and hypertension. Subsequently, adjustments were made for various confounders. In Model 1, adjustments were made for age and sex. In Model 2, additional adjustments were made for BMI, diabetes, hypertension, and LDL levels. In Model 3, further adjustments were made for diet and education level. Risk ratios (RR) and 95% confidence intervals (CI) were calculated for the binary outcome variables. All statistical analyses were performed using StataNow version 18.5. Baseline characteristics of the study population are summarized in Table 1.

**Table 1:** Baseline characteristics of patients with and without PPI intake. Most continuous variables were non-normally distributed. All continuous variables are presented as median (IQR). Categorical data are given as numbers (percentage) and compared using the chi-square test. All tests were two-sided, and a p-value of <0.05 was considered statistically significant.

	Total	No PPI	PPI	*p*-value
	*N* = 6153	*N* = 5685	*N* = 468	
Age	58 (52–66)	57 (52–65)	64 (56–70)	< 0.001
Age < 45 years	5% (337)	6% (324)	3% (13)	
Age 45–54 years	31% (1922)	32% (1847)	16% (75)	
Age 55–64 years	35% (2145)	35% (1981)	35% (164)	
Age 65–74 years	22% (1364)	21% (1209)	33% (155)	
Age ≥ 75 years	6% (385)	6% (324)	13% (61)	
Sex				0.049
Female	48% (2939)	47% (2695)	52% (244)	
Male	52% (3214)	53% (2990)	48% (224)	
BMI	27 (24–30)	26 (24–30)	28 (25–32)	< 0.001
BMI ≥ 30	24% (1455)	23% (1290)	35% (165)	
BMI 25 to < 29	42% (2567)	42% (2368)	43% (199)	
BMI < 25	35% (2131)	36% (2027)	22% (104)	
Hypertension	58% (3558)	57% (3213)	74% (345)	< 0.001
BP ≥ 140 or ≥ 90 mmHg	46% (2826)	45% (2581)	52% (245)	
BP intermediate	50% (3054)	50% (2845)	45% (209)	
BP < 120/80mmHg	4% (273)	5% (259)	3% (14)	
Diabetes (yes/no)	17% (1061)	17% (941)	26% (120)	< 0.001
HbA1c [%]	5.5 (5.3–5.8)	5.5 (5.2–5.8)	5.6 (5.4–5.9)	< 0.001
Creatinin [mg/dL]	0.9 (0.8-1.0)	0.9 (0.8-1.0)	0.9 (0.8–1.1)	0.11
Hemoglobin [g/dL]	14.6 (13.8–15.4)	14.6 (13.8–15.4)	14.4 (13.5–15.1)	< 0.001
MCV [fl]	87 (85–90)	87 (84–90)	87 (85–90)	0.73
Thrombocytes [×10³/µL]	230 (197–268)	230 (198–267)	230 (190–274)	0.47
Leukocytes [×10³/µL]	5.8 (4.9–6.9)	5.7 (4.9–6.9)	6.1 (5.1–7.2)	0.054
CRP [mg/dL]	0.2 (0.1–0.4)	0.2 (0.1–0.4)	0.3 (0.1–0.5)	< 0.001
Cholesterol	219 (191–248)	220 (192–248)	210 (181–245)	< 0.001
Cholesterol ≥ 240 mg/dL	32% (1937)	32% (1806)	28% (131)	
Cholesterol 200 to 239 mg/dL or treated	46% (2810)	45% (2561)	53% (249)	
Cholesterol < 200 & untreated	23% (1391)	23% (1303)	19% (88)	
LDL [mg/dL]	140 (115–167)	141 (116–167)	132 (109–164)	< 0.001
HDL [mg/dL]	56 (47–67)	56 (47–67)	53 (45–63)	< 0.001
Metabolic syndrome (yes/no)	77% (4741)	76% (4322)	90% (419)	< 0.001
Education				< 0.001
Lower education	39% (2217)	38% (1999)	52% (218)	
Medium education	53% (3019)	53% (2830)	45% (189)	
High education	8% (474)	9% (462)	3% (12)	
Smoking status				< 0.001
Never smoker	39% (1906)	39% (1787)	33% (119)	
Ex-smoker	38% (1886)	38% (1710)	48% (176)	
Active smoker	23% (1124)	23% (1056)	19% (68)	
Alcohol				< 0.001
No alcohol	15% (858)	14% (752)	24% (106)	
< 2 drinks/day	72% (4137)	73% (3871)	60% (266)	
≥ 2 drinks/day	14% (783)	13% (715)	15% (68)	
Red meat servings per week	2 (1–3)	2 (1–3)	2 (1–3)	0.074
Physical activity				< 0.001
< 1 h	18% (850)	17% (742)	27% (108)	
< 2 h	44% (2110)	44% (1932)	45% (178)	
< 3 h	29% (1392)	30% (1316)	19% (76)	
≥ 3 h	10% (461)	10% (428)	8% (33)	
Fastfood				0.13
Fastfood < weekly	81% (5012)	82% (4643)	79% (369)	
Fastfood ≥ weekly	19% (1141)	18% (1042)	21% (99)	
Vegetables				0.12
Vegetables ≥ daily	46% (2804)	46% (2607)	42% (197)	
Vegetables < daily	54% (3349)	54% (3078)	58% (271)	
Fruits				0.69
Fruits ≥ daily	40% (2458)	40% (2267)	41% (191)	
Fruits < daily	60% (3695)	60% (3418)	59% (277)	

BMI: Body Mass Index; BP: Blood Pressure; HbA1c: Hemoglobin A1c; MCV: Mean Corpuscular Volume; CRP: C-Reactive Protein; LDL: Low-Density Lipoprotein; HDL: High-Density Lipoprotein;

## Results

**Fig. 2:**
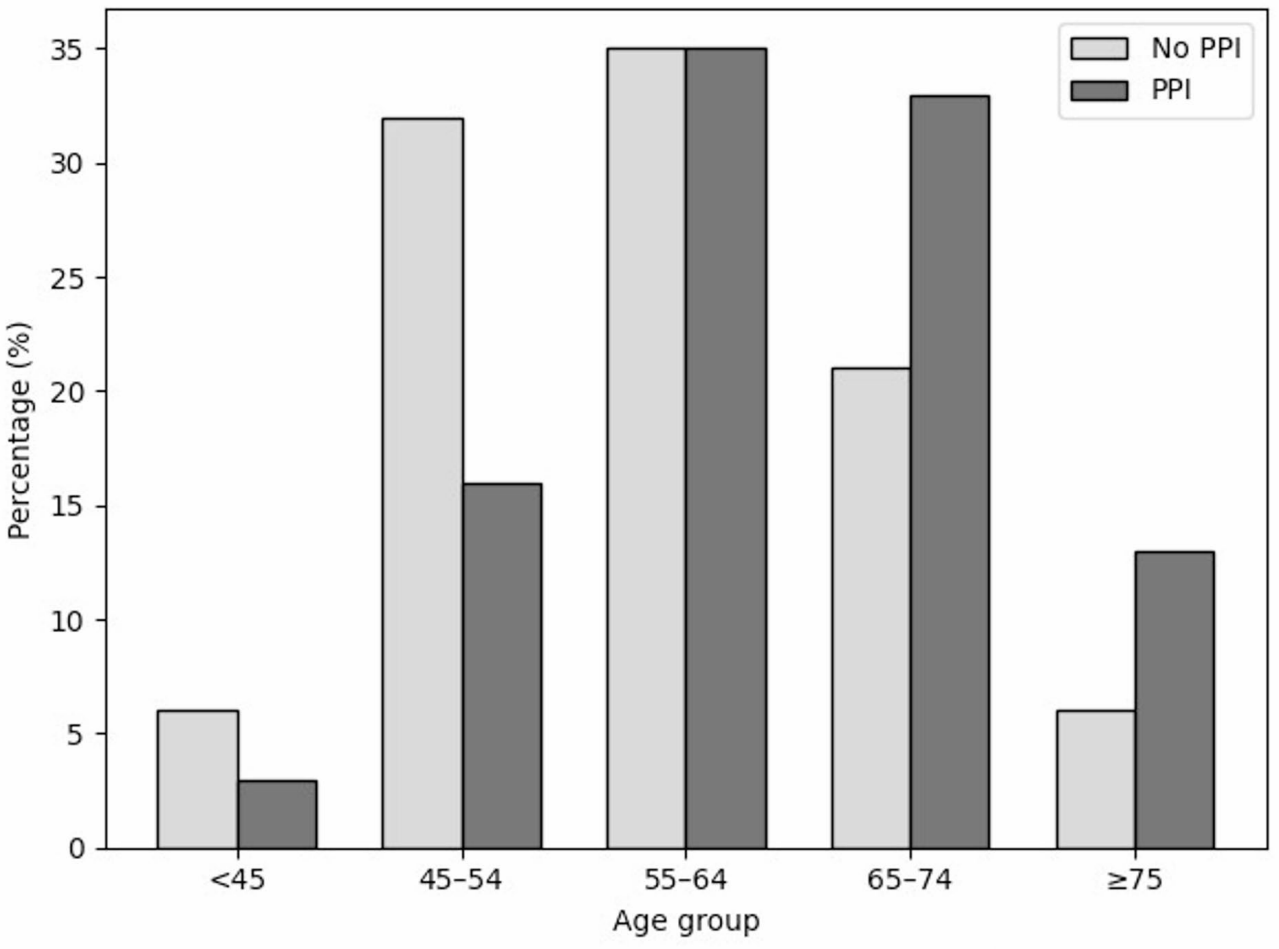
The percentage distribution of age groups among patients with and without PPI intake reveals distinct patterns. Among PPI users, older age groups are more prevalent, with a higher proportion of individuals aged 65-74 years (33%) and those aged 75 years and older (13%), compared to non-users, who have 21% and 6% in these respective age groups. Conversely, younger age groups, particularly those under 45 years and between 45-54 years, are less represented among PPI users (3% and 16%, respectively) compared to non-users (6% and 32%). This indicates that PPI use is more common in older patients, reflecting a trend where the likelihood of PPI use increases with age.

In this study involving 6153 individuals, 5685 (92%) were not on PPI therapy as part of their regular medication regimen at the time of presentation, whereas 468 (8%) were using a PPI. Significant differences were noted between PPI users and non-users, particularly in age, BMI, and the prevalence of diabetes, hypertension, and metabolic syndrome. PPI users were generally older, with a median age of 64 years (IQR: 56–70) compared to 57 years (IQR: 52–65) for non-users (*p* < 0.001), and a higher prevalence of older age groups was observed among them. (Fig. 2).

 In our study cohort, PPIs were more commonly used by women at the time of the examination (52%, *p* = 0.049). Additionally, PPI users had a higher median BMI (28 vs. 26, *p* < 0.001) and were more likely to have a BMI ≥ 30 (35% vs. 23%). They also had higher rates of hypertension (74% vs. 57%, *p* < 0.001) and diabetes (26% vs. 17%, *p* < 0.001), with slightly elevated HbA1c levels. The prevalence of metabolic syndrome was significantly higher among the PPI group (90% vs. 76%, *p* < 0.001). Furthermore, participants with PPI intake were more likely to have lower educational attainment (52% vs. 38%, *p* < 0.001) and exhibited differences in lifestyle factors, such as lower levels of physical activity. (Table 1) Diverticulosis was identified endoscopically in 37% (2,276) of all patients. Regarding the prevalence of diverticulosis, the rates were significantly higher in the PPI user group compared to non-users (48% vs. 36%, *p* < 0.001). This was observed regardless of the location of the diverticula. (Table 2)

**Table 2:** Percentage of patients with and without diverticulosis in the corresponding PPI category.

	Total	No PPI	PPI	*p*-value
	*N* = 6153	*N* = 5685	*N* = 468	
Diverticulosis	37% (2276)	36% (2052)	48% (224)	< 0.001
No diverticulosis	63% (3877)	64% (3633)	52% (244)	
Left sided diverticulosis	24% (1453)	23% (1314)	30% (139)	
Right sided diverticulosis	5% (280)	4% (246)	7% (34)	
Pandiverticulosis	9% (543)	9% (492)	11% (51)	

Consistent with the observed data trends, the univariate Poisson regression analysis revealed a statistically significant association between PPI use and the presence of diverticulosis (RR 1.326, 95% CI: 1.199–1.476, *p* < 0.001). Further analysis using a dose-comparative regression model revealed no significant differences between half standard dose and standard dose (half standard dose: RR 1.314, 95% CI: 1.143–1.511, *p* < 0.001 vs. standard dose: RR 1.338, 95% CI: 1.167–1.534, *p* < 0.001), suggesting no dose-dependent effect of PPI use on the prevalence of diverticulosis. Sensitivity analysis further explored this association across various subgroups. (Table 3).

**Table 3:** Sensitivity analyses for sex, age, obesity (BMI>30), diabetes and hypertension of PPI intake and diverticulosis.

Subgroup	Sensitivity analysis		
	RR	CI 95%	*p*-value
Sex			
Male	1.219	1.054–1.409	0.008
Female	1.463	1.271–1.683	< 0.001
BMI			
< 30	1.356	1.190–1.546	< 0.001
≥ 30	1.159	0.991–1.355	0.065
Age			
≤ 55a	1.210	0.860–1.705	0.274
> 55a	1.168	1.054–1.294	0.003
Diabetes			
No diabetes	1.562	1.202–2.030	0.001
Prediabetes	1.153	0.979–1.360	0.088
Diabetes	1.019	0.828–1.255	0.858
Hypertension			
No Hypertension	1.247	0.976–1.593	0.077
Hypertension	1.248	1.119–1.392	< 0.001

PPI use was significantly associated with diverticulosis in both males and females. Among individuals with a BMI < 30, PPI use was significantly linked to diverticulosis; however, this association was not significant in those with a BMI ≥ 30. In patients over 55 years of age, PPI use remained significantly associated with diverticulosis, whereas no significant association was observed in younger patients. Additionally, a significant association was found in individuals without diabetes, but not in those with prediabetes or manifest diabetes. PPI use was also significantly associated with diverticulosis in patients with hypertension.

In the multivariable adjustment analysis, the association between PPI intake and diverticulosis was examined across three models with increasing levels of adjustment for potential confounders. (Table 4)

**Table 4:** Sensitivity analyses for sex, age, obesity (BMI>30), diabetes and hypertension of PPI intake and diverticulosis.

Subgroup	Multivariable adjustment		
	RR	CI 95%	p-value
Model 1	1.119	1.012–1.238	0.029
Model 2	1.014	0.904–1.138	0.809
Model 3	1.045	0.916–1.193	0.511

In model-1, which adjusted for age and sex, PPI use was significantly associated with diverticulosis (RR = 1.119, 95% CI: 1.012–1.238, *p* = 0.029). However, in model-2, which additionally adjusted for BMI, diabetes, hypertension, and LDL levels, the association was not significant (RR = 1.014, 95% CI: 0.904–1.138, *p* = 0.809). Similarly, in model-3, which included further adjustments for dietary factors and education level, the association remained non-significant (RR = 1.045, 95% CI: 0.916–1.193, *p* = 0.511). These results suggest that the initially observed association between PPI use and diverticulosis in the univariate analysis and in model-1 is attenuated after adjusting for key confounders, indicating that the relationship may be influenced by other factors rather than a direct effect of PPI use alone.

## Discussion

In this cohort of 6153 asymptomatic participants, examined as part of a colorectal cancer prevention project, valuable insights were gained into the association between PPI use and the prevalence of colonic diverticulosis. While an initial association between PPI intake and diverticulosis was observed in univariate analysis, no statistically significant association was found after adjusting for potential confounding factors.

PPI users in this study were generally older, had higher BMI, and exhibited a greater prevalence of metabolic syndrome, hypertension, and diabetes compared to non-users. Explaining this statistical result, in which no association is observed after adjusting for confounders, appears to be a clustering of PPI use among patients who potentially have an increased risk for diverticula formation due to other underlying causes.

It is now widely recognized that the occurrence of diverticula significantly increases with advancing age^20^. A severe manifestation of diverticulosis has also been observed with increasing age^16,30^, which is attributed to structural changes in the colonic wall as part of the aging process^20^. Regarding obesity, not only has a higher BMI been associated with an increased occurrence and greater severity of diverticula^30,31^, but an association has also been observed with increased accumulation of abdominal visceral and subcutaneous fat, even in individuals with a BMI below 25, indicating normal weight^32^. Accordingly, an association with increased waist-to-hip ratio^33^ and higher waist circumferences^34^ has also been described. Furthermore, an accumulation of diverticula was also observed in association with metabolic syndrome, taking into account the overall limited availability of data^35^. There is also a clear association between arterial hypertension and diverticulosis, particularly when the hypertension is poorly controlled or untreated^20,36^. In contrast, studies on the relationship between diabetes mellitus and diverticulum formation yield inconclusive results^20,37^. Moreover, a low level of education, which in our study was associated with higher rates of PPI use, was independently linked to the formation of diverticula^38^.

The co-occurrence of these factors highlights the necessity of careful adjustment for potential confounders when investigating associations between PPI use and health outcomes.

Up to now fecal and mucosa-associated microbiome in diverticular disease do not show clear alterations related to diverticular formation or disease progression, with results being inconsistent across different studies^22,23^. The current body of evidence regarding the association between PPI use and the occurrence of diverticula is extremely limited. Several studies have examined the relationship between PPI use and diverticulitis, a potential complication of the asymptomatic diverticulosis.

In a multicenter study conducted by Sbeit et al., a statistically significant association between diverticulitis and PPI use was observed, even after adjusting for confounding factors^21^. However, it is important to note certain limitations, particularly the age and pre-existing conditions of the study population, as well as the retrospective design of the study. Conflicting results were reported in a case-control study from Taiwan, based on a National Health Insurance Research Database, which found that the use of PPIs did not increase the risk of diverticulitis^39^. It should be noted, however, that the predominantly right-sided diverticular formation in the Asian population, which differs from the pattern observed in Western populations, must be taken into account. This divergence, likely influenced by distinct pathophysiological mechanisms, could explain the contrasting associations. Tursi et al. additionally demonstrated that the use of PPIs was associated with the severity of diverticular disease, as measured by the DICA classification^20^. It should be mentioned that this association was observed only in the univariate analysis and did not demonstrate statistical significance in the multivariate analysis.

Several studies suggest that proton pump inhibitors (PPIs) alter the gut microbiome by influencing the composition of gut flora and moderately reducing its diversity^6^. This has been linked to various gastrointestinal disorders, including increased intestinal permeability, lower gastrointestinal bleeding, small intestinal bacterial overgrowth, Clostridium difficile-associated diarrhea, inflammatory bowel diseases, and biliary system disorders^40^. However, the evidence remains limited and sometimes contradictory, as many findings are based on retrospective analyses or animal models with methodological weaknesses. In particular, the role of the microbiome in potential PPI-induced digestive disorders is not yet well understood.

Importantly, diverticulosis is a chronic condition that develops over prolonged periods. Any potential effect of proton pump inhibitors - hypothetically mediated through long-term mechanisms such as sustained alterations of the gut microbiome - would therefore be expected to depend on cumulative exposure. In this context, current regular PPI use in the present analysis should be interpreted as a proxy for long-term PPI exposure, which cannot be precisely quantified within the constraints of a cross-sectional study design. Therefore, high-quality longitudinal studies are needed to better investigate the underlying mechanisms and validate current findings.

This analysis is strengthened by a large participant cohort and careful consideration of potential confounders. Nevertheless, several limitations warrant discussion. First, the cross-sectional design captures exposure and outcome at a single time point, precluding causal inference and assessment of temporal relationships, and leaving residual confounding possible. Second, information on the indication for proton pump inhibitor (PPI) use, cumulative treatment duration, and specific PPI compound was not systematically collected. Given established effects of PPIs on the gut microbiota and variability in acid-suppressive potency across agents, this limitation prevented duration-dependent, compound-specific, and mechanistic analyses. The study population was drawn from an asymptomatic colorectal cancer screening setting, which may introduce selection and information bias. Although symptom-driven referral to colonoscopy was largely minimized by the screening context, abdominal discomfort, pain, or generally increased health care–seeking behavior may have acted as upstream confounders influencing both PPI prescription and health care utilization. Detailed data on gastrointestinal symptoms, indications for PPI therapy, and individual patterns of medical surveillance were unavailable and therefore could not be explicitly accounted for, raising the possibility of residual confounding related to unmeasured aspects of medication use or health care exposure. The single-center design further limits generalizability, as regional patient characteristics and environmental factors may not reflect those of broader Western populations. In addition, the open and opportunistic nature of the screening program precluded determination of an exact participation rate within the eligible source population. Although demographic characteristics were contextualized using regional population statistics, overregional patient flow represents an additional limitation. Finally, the study was not designed to investigate underlying biological mechanisms, leaving cellular and molecular pathways unexplored.

Future research should prioritize longitudinal designs to clarify temporal and causal relationships between diverticulosis and PPI use. Complementary basic and translational studies examining cellular and molecular mechanisms will be essential to advance mechanistic understanding.

## Conclusion

In this large, asymptomatic colorectal cancer screening cohort, an initially observed association between proton pump inhibitor use and the prevalence of diverticulosis was not sustained after adjustment for demographic, clinical, and lifestyle-related confounders. These findings indicate that PPI use itself is not independently associated with diverticulosis, and that the higher prevalence observed among PPI users in unadjusted analyses is likely explained by underlying factors such as older age, increased cardiometabolic risk, and socioeconomic characteristics.

Accordingly, this study does not provide evidence to support a causal role of PPI therapy in the development of colonic diverticulosis. Future longitudinal studies with detailed assessment of PPI exposure duration and indications are required to further clarify temporal relationships and potential mechanisms.

## Data Availability

Data is provided within the manuscript.
